# Institutional ELN/LIMS deployment: Highly customizable ELN/LIMS platform as a cornerstone of digital transformation for life sciences research institutes

**DOI:** 10.15252/embr.201949862

**Published:** 2020-02-27

**Authors:** Nicolas Argento

**Affiliations:** ^1^ EPFL Lausanne Switzerland

**Keywords:** Methods & Resources, S&S: Ethics

## Abstract

The systematic recording and management of experimental data in academic life science research remains an open problem. EPFL engaged in a program of ELN/LIMS deployment 6 years ago, encountering a host of fundamental questions at the institutional level and in each single laboratory. Here, based on our experience we aim to share with research institute managers, PIs, and any scientists involved in an ELN/LIMS deployment, helpful tips and tools to surround yourself with the right people and the right software at the right time. In this article we describe the resources used, the challenges, key success factors, and the results obtained at each phase of our project. Finally, we discuss the current and next challenges and how our experience leads us to support the creation of a new position in the research groups: the laboratory data manager.

## Graphical abstract

Scientists have been reluctant to use electronic notebooks and lab data management systems. Their wider adoption requires institutional planning, IT support and convincing lab members of the advantages and benefits.

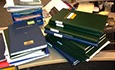

Research tools in the life sciences are continuously evolving and improving, and scientists have always been eager to use the latest equipment. Ironically though, their main method of recording and managing experimental data has remained largely the same for centuries (Fig 1): The laboratory notebook is still the main method of record‐keeping. The adoption of electronic laboratory notebooks (ELNs—Box 1) in academic laboratories has been slow—if laboratories have actually shown any interest at all. Their implementation necessitates institutional support 1, and despite much discussion of ELNs in the literature 2, 3, success stories and recipes for their deployment remain scarce 4, 5, 6. Moreover, although ELNs can improve efficiency in data capturing and re‐use, they lack the features to rigorously document data critical for experimental reproducibility, such as sample traceability and standard operating procedures (SOP). These features are, however, part of another tool for data management called the Laboratory Information Management System (LIMS—Box 1).

**Figure 1:**
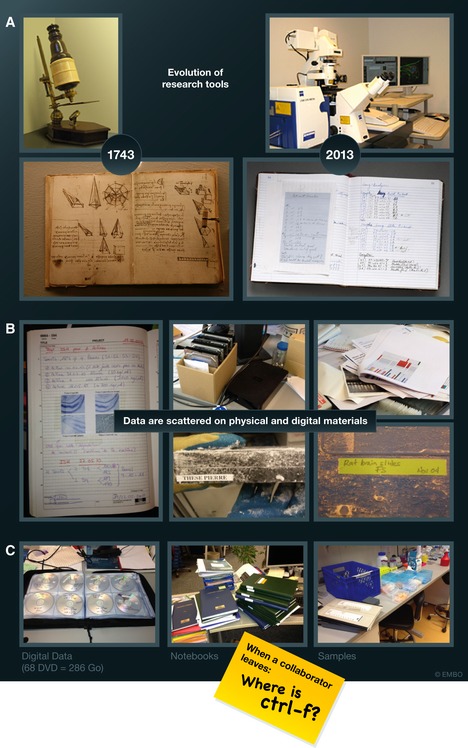
Challenges of traditional record‐keeping in modern science (A) Comparison of research instruments and laboratory records from the 1700s and 2000s. While the development of research instruments has driven progress, laboratory record‐keeping is almost unchanged. (B) Data are distributed across multiple locations and media, including notebooks, printed material, physical samples, disks, hard drives, servers and cloud storage. (C) Locating specific data is difficult as the location may be unknown, and the media may be difficult to search.

**Box 1. ELN and LIMS main features in research institutions**

**Table:** 

**LIMS, a digital tool for** Sample management (plasmids, virus, antibodies, chemicals databases) Stock management Workflow templates Standard operating procedure management Laboratory equipment inventory Equipment integration for direct data acquisition	**ELN, the digital substitute to paper notebook** Research work traceability Knowledge transmission Legal: intellectual property and patents

In order to encourage adoption of ELNs at the institutional level, EPFL started a dedicated program for ELN/LIMS deployment 6 years ago that involved institute managers, PIs, and scientists at all levels. Here, we share the challenges, key success factors, and the results obtained at each phase of our project (Fig 2). Finally, we discuss the current and upcoming challenges and how our experience led us to support the creation of a new position in research groups: the laboratory data manager.

**Figure 2:**
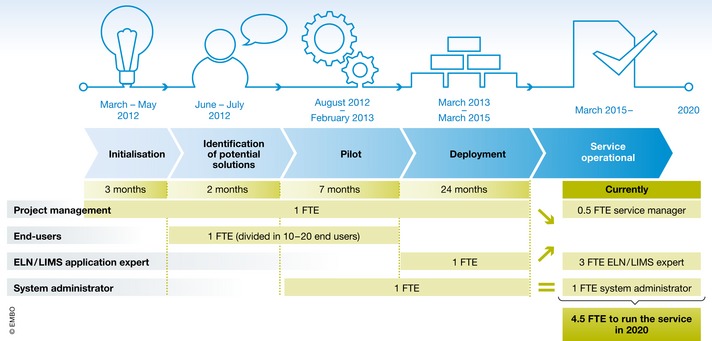
Institutional ELN/LIMS project macro‐planning Project phases are indicated in blue underneath a timeline from March 2012, and associated human resources in FTE (full‐time equivalent) for each period are indicated in green (see text for details).

## Initiating the project

By definition, an institutional LIMS and ELN project involves management, PIs, scientists at all levels, and the institute's IT department; the steering committee for our project therefore included the Vice President of Research, who is responsible for scientific information, and the Vice President of Information Systems, who is responsible for IT governance and IT core services on campus. Representing the main users, the Life Sciences Faculty's Dean chaired the committee. This executive body defined the overall goals: to rationalize laboratories’ efforts, reduce waste of time and money, reduce loss and enable re‐use of data, improve the reproducibility of experiments, and facilitate data sharing for collaborative projects.

By definition, an institutional LIMS and ELN project involves management, PIs, scientists at all levels and the institute's IT department…

The steering committee hired a project manager to coordinate the interests and requirements of the multiple stakeholders from distant fields, which made this project as complex as it was fascinating. To make sure that the introduction of an ELN/LIMS service was suitable for those affected, we involved a panel of scientists. The project manager also played the role of a “business analyst” by meeting and surveying 25 laboratories whose needs and demands were synthesized in a weighted wish list that, along with legal requirements, helped to choose the ELN/LIMS solution. For instance, our institutional rules and laws about privacy and the use of human data prevented us from using a cloud‐based solution.

## Identification of a suitable ELN/LIMS platform

**Figure 3:**
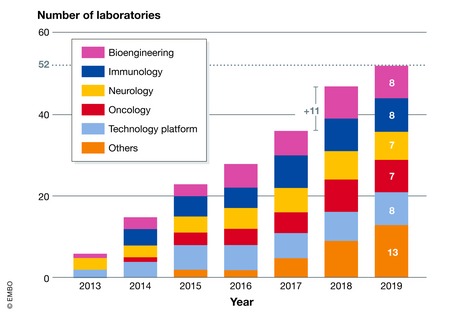
Laboratory participation by institute Number of laboratories participating in the ELN/LIMS project at EPFL from each Institute in the School of Life Sciences listed since the project's inception in 2019. Others refers to laboratories at EPFL, but outside life sciences. Note the largest increase in laboratory participation in 2018 (see text for details).

The initiation phase revealed that the project required more than just picking an off‐the‐shelf ELN. Our key users were active in immunology, oncology, neurology, and bioengineering with correspondingly diverse approaches and demands; in addition, technology platforms showed a strong interest; their routine workflows required additional administrative support (Fig 3). More generally, the analysis highlighted difficulties in managing data related to laboratory SOPs and samples, which are crucial for data reusability and experimental reproducibility in biomedical research 7. Samples and experiments with human data require more sophisticated privacy management, a requirement that is increasing with the rise of personalized medicine. Feedback from laboratory staff also indicated that integration with third‐party information systems would be of added value for everyday work. Typically, importing data from an animal facility was relevant to the experimental laboratories, while integration with work request forms and integration with the billing system were key features for technology platforms. The issue of authentication was raised by laboratories and IT staff, for ease of use for the first group and security for the second group. Involving the IT department from the beginning also prevented us from taking obvious wrong directions in terms of technical choice. A striking example of their input was highlighting the heterogeneity of the scientists’ personal computers in the research institute, which made the choice of a Web‐based solution almost mandatory, rather than installing software on each computer. However, the most interesting technical question was how to manage the diversity of needs. And the most important question for scientists was how to preserve creativity and freedom of research without introducing new burdens and hassles.

Individual laboratories using our services remain architects of their own information system so as to preserve and maintain freedom and creativity. An ELN typically has highly standardized features, but new software technologies allow the creation of highly customizable databases and graphical user interfaces. The software we chose uses visual, declarative techniques instead of programming to enable fast, iterative, collaborative, and tailored implementation. Applications can be rapidly modified and maintained centrally.

Involving the IT department from the beginning also prevented us from taking obvious wrong directions in terms of technical choice.

Mastering such a powerful toolbox required the appointment of skilled people and good practice of implementation. This investment is counterbalanced by the possibility of including a wide range of data, development of homemade features, and integration with other information systems—finance, work request forms, and so on. It also opens the possibility for compliance with ISO 9001 or FDA 21 CFR Part 11 standards that are required by some technology platforms. Those concerns are not industry's preserve and can help to foster reproducibility also in the life sciences 8, 9.

## Implementation

**Figure 4:**
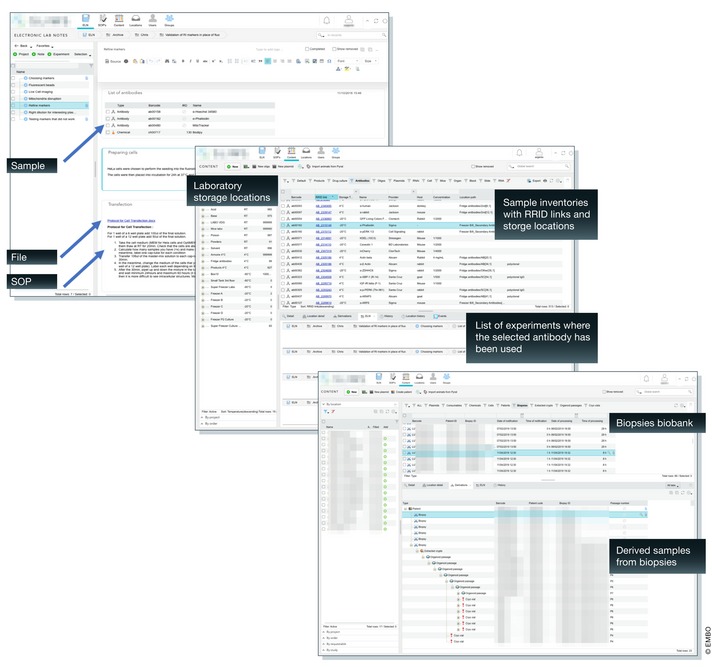
Typical usage of the ELN/LIMS platform (A) ELN with scientist's notes, samples linked to the laboratory inventories, files, and procedures showing how we solved the initial issue illustrated in Fig 1C. (B) Laboratory sample inventories viewed with associated physical storage locations and links to ELN entries. (C) Example of a biopsy biobank and list of all derived crypts, organoids, and cryovial samples from one single biopsy.

To accurately assess the personnel and skills needed to install and configure the platform and then train the staff required a dedicated budget to run a pilot phase. This budget covered a 6‐month license fee for the ELN/LIMS platform and a system administrator along with training and support. Through this pilot, five volunteer laboratories began to configure and use the ELN/LIMS platform. At the end, all stakeholders validated the choice of the solution over the short and long term, and the steering committee approved deployment at a larger scale. Fig 4 shows examples of the current typical usage of the ELN/LIMS platform.

The same staff then organized the deployment. A dedicated ELN/LIMS platform engineer was hired to help laboratories to get the most from the customizable platform (Fig 2). Such an ELN/LIMS expert must have strong IT competencies, project management skills, and a good general knowledge in research to efficiently communicate with the scientific staff. More specifically, the engineer developed and optimized the work methodology to ensure sustainable growth from a technical and scientific point of view. The announcement of the Swiss National Science Foundation (SNSF) to make a data management plan mandatory for all grant applications from October 2017 created a peak of demand for our services in 2018, when 10 laboratories voluntarily started ELN/LIMS deployment (Fig 3) (http://www.snf.ch/en/researchinFocus/newsroom/Pages/news-170306-towards-open-research-data.aspx). Nonetheless, laboratories were not forced to use ELN/LIMS and it still remains a PI's decision to use the platform. In the following sections, we present the step‐by‐step approach to deploy the platform in a laboratory. From our experience, each step contributes to successful and sustained ELN/LIMS adoption and use.

Individual laboratories using our services remain architects of their own information system so as to preserve and maintain freedom and creativity.

Each ELN/LIMS platform deployment is managed as a separate sub‐project, since the information systems are tailor‐made for each laboratory. The deployment phase starts with informal discussions between the ELN/LIMS application expert and the laboratory management to demonstrate the offered services. This introduction aims to confirm that the tool could support the laboratory's objectives. If so, the list of objectives is formalized and validated by the project sponsor, the PI in this case. Objectives are prioritized and have an appointed reference person (Box 2). Roles and responsibilities must be clarified.

**Box 2. Example of research laboratory requirements sorted by decreasing priority**

- Configure and adopt ELN/LIMS for all laboratory members
- Create a standard operating procedure (SOP) and simple operating procedure library accessible for everyone
- Centralize antibody database
- Centralize plasmid database
- Centralize chemical database
- Manage the laboratory storage location (freezer, cabinet, etc.)
- Define uniform identification of locations and sample thanks to label printers

## The stakeholders and their roles in the deployment

The PI is the sponsor, who initiates the project and assumes overall responsibility. He or she usually delegates the work to appropriate staff members. Along the deployment, the PI can be asked to take decisions on proposals.

ELN/LIMS end users are the laboratory staff and PIs and their active participation in the deployment and their remarks and comments are crucial for setting up a tool that fits their needs and habits.

**Figure 5:**
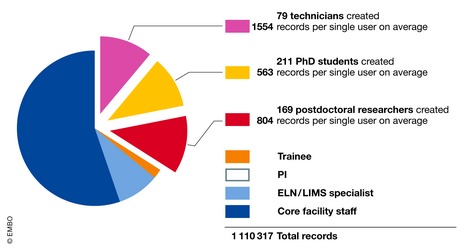
Record creation by institutional role Pie chart describes the proportion of records (file, sample, or experiment) created as a function of the institutional role of the person creating the record at EPFL. Note that records where the institutional role was not available are not shown, amounting to < 0.4% of the total. The records created by PIs are not visible at this scale (see text for details).

Laboratory referents take the lead and responsibility to fulfill the aims delegated by the PI. Initially, this task was given to newly arrived PhD students, but we soon realized that a thorough knowledge of the laboratory operation is required for efficient implementation. This role should therefore be given to experienced scientists or technicians; the latter often have associated laboratory planning tasks and are less likely to leave than scientific staff. Those considerations make them more prone to act as a locomotive in adopting the ELN/LIMS platform. This view is supported by the raw record creation quantity according to the staff position (Fig 5).

The ELN/LIMS application expert is responsible for configuring the system according to the laboratory referents’ requests and to advice about best practices; they translate the identified needs into configuration and code. A strong know‐how in ELN/LIMS customization and best practices and general knowledge about the scientific field are tremendously important. By working in several laboratories, our ELN/LIMS expert team developed strong skills in laboratory data management and project management that the typical laboratory does not have. Consequently, they currently work as deployment project manager, whereas ideally, their role should be restricted to supporting the workflows of the adopting laboratory, not imposing (well‐intentioned) ideas from the outside.

ELN/LIMS end users are the laboratory staff and PIs, and their active participation in the deployment and their remarks and comments are crucial for setting up a tool that fits their needs and habits. While sample and SOP management are usually easily adopted, the use of the ELN is trickier as it offers a large range of possibilities to organize projects and experiments compared with paper notebooks. This can make adaptation frustrating and can take weeks; indeed, some laboratories decided not to use the ELN or only a part of the staff adopted it. We have also seen a few laboratories abandon the ELN. Close support during the first week of usage and regular communication are necessary to reduce teething troubles. Here, the normal turnover of scientific staff in research laboratories can be used as an opportunity. It is problematic to ask a post‐doctoral researcher or a PhD student to change their data management tools and habits in the middle of their project, whereas new recruits can start fresh with the ELN/LIMS system.

… data management should be driven by science, and not vice versa, which is one of the reasons we propose a “laboratory data manager” for managing sensitive or crucial data.

## From needs analysis to conception and realization

**Figure 6:**
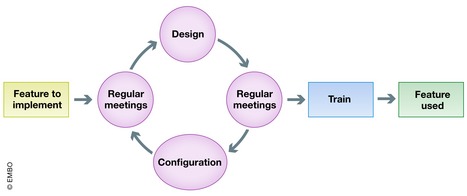
Agile cycle adapted to ELN/LIMS deployment in life sciences research laboratory Flow chart of process and decisions in ELN/LIMS deployment used at EPFL. Note the agile cycle shown in purple for rapid adaptation of the platform to the needs of each laboratory.

In this phase, the ELN/LIMS expert needs work as closely as possible with the researchers to translate laboratory context, culture, and workflow into the configuration. According to our experience, the conception must be an incremental process. This process is common practice in the software industry's “Agile” methodology based on continuous small deliveries and short daily meetings 10. During deployment, we organize regular meetings to discuss the ELN/LIMS platform configuration and design and, depending on the test performed by the referent, further adapt the configuration for the next meeting (Fig 6). The cycle is repeated until the result is sufficiently convincing to validate the deployment of this feature and corresponding training.

**Figure 7:**
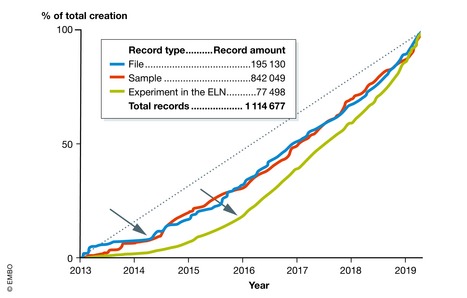
Evolution of raw record creation in research laboratories Increase in the number of file, sample, and experiment records is plotted, normalized to the total number for each class at the time of writing, over the period of the ELN/LIMS project at EPFL. Note the inflection points in file and sample creation in mid‐2014, and in experiments at the beginning of 2016 (see text for details).

For almost 5 years, we have been serving the laboratories deployed along with the project and new volunteer laboratories. The raw record creation (files and samples) in the platform accelerated in mid‐2014 when technology platforms joined the system (Fig 7). The number of created experiments in the ELN (green) has grown more rapidly since 2016, reflecting improvement in training, communication between stakeholders, and continuous upgrades.

Although the service is hosted at the School of Life Sciences IT department, the platform has been requested and used by biologists from other faculties on the campus (Fig 3). In early 2020, three full‐time ELN/LIMS application expert staff serve 52 laboratories. This team is composed of people with heterogenous competencies from bioengineering and chemistry to computer sciences and IT management. In addition, a system administrator FTE operates the servers and data storage, while 0.5 FTE ensures the service management. The whole service requires 4.5 FTE positions (Fig 2) to guarantee his sustainability and evolution. They ensure day‐to‐day support for the deployments and users’ trainings while coordinating and performing the regular maintenance of the infrastructure. A frequently overlooked but important and time‐consuming task is to ensure platform upgrades, which has a key impact on user experiences and data integrity.

One challenge that remains […] is the slow adoption of the ELN by research staff, partly because the ELN is still a work in progress, partly because old working habits are slow to change.

One of our ELN/LIMS configurations is used by an “industrial” technology platform that sterilizes glass, decontaminates waste, and prepares standard solutions. They are ISO 9001 certified, and their data management is audited every year. As we operate the major part of their information systems, we are audited too and have put a quality system in place for maintenance and data backup. Generally, technology platforms are natural customers of such services. They run more standardized experiments with higher requirements for traceability. Figure 5 shows that they are the main record producer in the ELN/LIMS platform.

## The laboratory data manager

**Figure 8:**
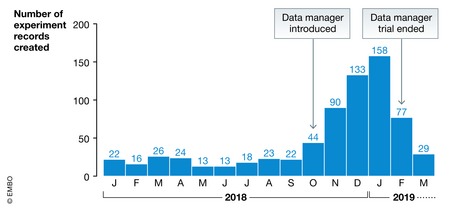
Increased experiment record creation with the introduction of a laboratory data manager The number of experiment records created in the ELN per month in 2018–2019 from a single laboratory at EPFL, displayed as a bar graph on a timeline, and annotated with the introduction and departure of a trained data manager in the laboratory.

We experienced that ELN/LIMS application experts were regularly asked by laboratory referents to organize their data management. However, data management should be driven by science, and not vice versa, which is one of the reasons we propose a “laboratory data manager” for managing sensitive or crucial data. Obviously, the particular skills required for a data manager in cutting‐edge research is a challenge. As a trial, we placed a properly skilled data manager into one of our research groups for a couple of months. As shown in Fig 8, it had an immediate impact on the amount of data collected in the ELN and enabled the laboratory to review their data management practices. We do not have information about the quality of the data produced, but we expect improvement at this level, and we anticipate that the publishing process could also be accelerated owing to better reusability of data inside the laboratory. A laboratory data manager would apply general institutional or research‐specific policies and good practices, and convert general infrastructure into daily practical solutions that fit the local needs. Being integrated in the laboratory is necessary to legitimize changing practices while maintaining the flexibility and freedom required by science. A data manager would not necessarily be a full‐time job, depending on the laboratory's activity.

In summary, we believe our institutional project showed that the ELN/LIMS platform helps to capture and record data, and helps to make the data to become Findable, Accessible, Interoperable, and Reusable (FAIR). The reusability criterion implies quality improvements that are more dependent on data management practice than on the available electronic tools.

One challenge that remains for the success of data management is the slow adoption of the ELN by research staff, partly because the ELN is still a work in progress, partly because old working habits are slow to change. Furthermore, managing huge files—genomic data or microscopy images—remains a major challenge, as is archiving the data collected by the ELN/LIMS systems. Finally, integration with other elements of the institutional information system is technically possible, but requires governance of business applications, interface development, and resources for maintenance. Amidst all of these challenges, we strongly believe funding the position of data managers remains a priority. In the end, it is the daily practice of the scientists that will drive and sustain the digital transformation of the life sciences.
